# The Role of Fluorine-18-Fluorodeoxyglucose Positron Emission Tomography in Aggressive Histological Subtypes of Thyroid Cancer: An Overview

**DOI:** 10.1155/2013/856189

**Published:** 2013-04-09

**Authors:** Giorgio Treglia, Salvatore Annunziata, Barbara Muoio, Massimo Salvatori, Luca Ceriani, Luca Giovanella

**Affiliations:** ^1^Department of Nuclear Medicine and Thyroid Centre, Oncology Institute of Southern Switzerland, Via Ospedale 12, 6500 Bellinzona, Switzerland; ^2^Institute of Nuclear Medicine, Catholic University of the Sacred Heart, Largo Gemelli 8, 00168 Rome, Italy; ^3^School of Medicine, Catholic University of the Sacred Heart, Largo Vito 1, 00168 Rome, Italy

## Abstract

Aggressive histological subtypes of thyroid cancer are rare and have a poor prognosis. The most important aggressive subtypes of thyroid cancer are Hürthle cell carcinoma (HCTC) and anaplastic and poorly differentiated carcinoma (ATC and PDTC). The American Thyroid Association recently published guidelines for the management of patients with ATC, but no specific guidelines have been done about HCTC. We performed an overview of the literature about the role of Fluorine-18-Fluorodeoxyglucose positron emission tomography or positron emission tomography/computed tomography (FDG-PET or PET/CT) in aggressive histological subtypes of thyroid cancer. Only few original studies about the role of FDG-PET or PET/CT in HCTC, PDTC, and ATC have been published in the literature. FDG-PET or PET/CT seems to be useful in staging or followup of invasive and metastatic HCTC. FDG-PET or PET/CT should be used in patients with ATC in initial staging and in the followup after surgery to evaluate metastatic disease. Some authors suggest the use of FDG-PET/CT in staging of PDTC, but more studies are needed to define the diagnostic use of FDG-PET/CT in this setting. Limited experience suggests the usefulness of FDG-PET or PET/CT in patients with more aggressive histological subtypes of DTC. However, DTC presenting as radioiodine refractory and FDG-PET positive should be considered aggressive tumours with poor prognosis.

## 1. Introduction

Aggressive histologic subtypes of thyroid cancer are less frequent and have a worse prognosis than well-differentiated thyroid carcinoma (DTC). Most important aggressive subtypes of thyroid cancer are Hürthle cell carcinoma (HCTC) and anaplastic and poorly differentiated carcinoma (ATC and PDTC).

HCTC was firstly considered as subtypes of DTC. Now, it is included in aggressive histologic subtypes, because of its biological behaviour. ATC could be a de novo tumour or arising from dedifferentiation of DTC. During this process, thyroid cancer could be found in an intermediate differentiation pattern, classified as PDTC. Both ATC and PDTC have a poor prognosis and efficient diagnostic tools are needed to improve survival.

After those about DTC and medullary thyroid cancer (MTC) [1, 2], American Thyroid Association (ATA) recently published guidelines for the management of patients with ATC [3]. No specific guidelines have been done about HCTC.

Fluorine-18-Fluorodeoxyglucose (FDG), a glucose analogue, is the most used positron emission tomography (PET) tracer in oncology. Although FDG-PET and PET/CT have a moderate sensitivity for early-stage, well-differentiated thyroid malignancy [4], they are currently used in DTC, particularly in postthyroidectomy patients with high serum thyroglobulin (Tg) levels and a negative radioiodine whole-body scan, as prognostic tool in patients with metastases, for the measurement of posttreatment response, and as selection tool in patients not eligible to radioiodine therapy [5–7]. FDG-PET or PET/CT is also used in recurrent MTC and in thyroid nodules with indeterminate or nondiagnostic fine needle aspiration biopsy (FNAB) [5].

During dedifferentiation process (from DTC to ATC), an inverse relationship between radioiodine (I-131) and FDG uptake in thyroid cancer cells was observed (the so-called flip-flop phenomenon) [8]. A recent study highlights that thyroid cancer dedifferentiation is characterized by glucose transporters (GLUT1) upregulation and reduced expression of sodium-iodide symporter (NIS) [9]. This is the rationale to propose FDG-PET or PET/CT as efficient diagnostic tools in ATC, PDTC, and other aggressive subtypes of thyroid cancer.

The aim of this paper is to perform an overview of the literature about the role of FDG-PET or PET/CT in aggressive histological subtypes of thyroid cancer.

## 2. FDG-PET in Hürthle Cell Thyroid Carcinoma (HCTC)

About 3.6% of thyroid cancers are HCTC [10]. Initially, HCTC was included in DTC group, but it has a different oncogenic expression and is now considered as different histological and clinic disease [11].

HCTC has a 10-year disease-free survival of 40% and mortality of 51% [12], worse than DTC. In fact, HCTC is associated with a high risk of distant and lymph nodal metastases having a worse prognosis compared to DTC. 

Only few studies about the role of FDG-PET or PET/CT in HCTC have been published in the literature. Some authors suggested a good sensitivity of FDG-PET in HCTC [13, 14] and Hürthle cell adenoma [15]. Overall, HCTC seems to be unable to concentrate I-131, but it is an FDG-avid tumour.

Pryma et al. [16] studied 44 patients with HCTC. There were 24 positive and 20 negative FDG-PET scans giving a sensitivity of 95.8% and a specificity of 95%. FDG-PET demonstrated a good diagnostic accuracy in HCTC patients. Furthermore, a high FDG uptake was demonstrated to be a negative prognostic factor. These authors suggested that FDG-PET could be indicated in patients with HCTC in postoperative staging and as followup in patients with an increase of Tg or recurrent disease [16].

Lowe et al. [17] studied 14 FDG-PET scans in patients with HCTC. PET findings were positive in all but 1 of patients with known disease, with a sensitivity of 92%. Moreover, in 7 out of 14 PET scans, a disease not diagnosed by other techniques was demonstrated. In 7 patients, therapy was changed after FDG-PET. So, these authors concluded that FDG-PET improves staging and disease management in patients with HTCT [17].

Plotkin et al. [18] evaluated 17 HCTC with FDG-PET. In subgroup A, patients with an elevated Tg level were included (*n* = 13), and in 10 cases PET scans were true positive. In subgroup B, patients with a suspect morphologic imaging were included (*n* = 4), and PET scans were true negative in three cases. Only one false positive was found in each group. Overall, FDG-PET demonstrated a sensitivity of 92%, a specificity of 80%, a positive predictive value of 92%, a negative predictive value of 80%, and an accuracy of 89% in HCTC [18].

**Figure 1:**
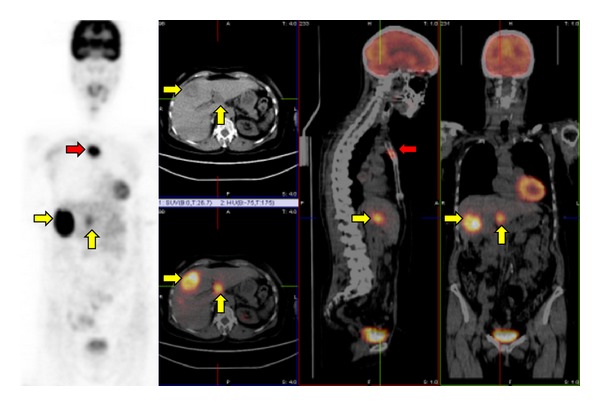
FDG-PET/CT in a 68-year-old female previously operated for HCTC showing the presence of sternal (red arrows) and liver metastases (yellow arrows).

Overall, FDG-PET or PET/CT seems to be useful functional imaging methods in initial staging or restaging of HCTC (Figure 1), presenting high diagnostic accuracy in this setting (Table 1).

## 3. FDG-PET in Anaplastic Thyroid Carcinoma (ATC)

ATC is a rare and aggressive tumour, representing less than 5% of all thyroid carcinomas and originating by thyroid follicular cells (as DTC). ATC is often diagnosed in older patients and usually has a rapid growth and an extensive local invasion [21–23]. Three main histological subtypes of ATC are reported: spindle cell, pleomorphic giant cell, and squamous cell subtype [3]. In over 70% of the patients the tumour infiltrates surrounding tissues, and median survival time is about 6–8 months [22]. In differential diagnosis, it is important to distinguish ATC and PDTC. In fact, proportion of ATC, PDTC, or DTC characterizing the thyroid tumour can change prognosis and clinical management [3].

This aggressive thyroid tumour is not able to uptake iodine and to produce Tg [19, 20, 24, 25]. Conversely, ATC has a high glucose metabolism and high FDG uptake [5, 9]. ATA recently published guidelines for management of patients with ATC [3]. ATA recommended FDG-PET and PET/CT in evaluating metastatic patients, especially bone lesions. Moreover, FDG-PET may be useful in distinguishing ATC from DTC metastases because of the higher FDG uptake of ATC. Other indications described about FDG-PET or PET/CT in ATC were resectability evaluation and followup, with a higher sensitivity than CT alone. FDG-PET is also recommended 3–6 months after therapy in patients with no disease or in persistent structural disease as a guide to therapy [3].

Few original studies have been published about the role of FDG-PET in ATC (Table 1).

Poisson et al. [19] studied 20 consecutive ATC patients with FDG-PET/CT for initial staging and during followup. Authors analysed progression on imaging followup (CT or PET/CT). Per lesion, organ, and patient analysis have been done. In univariate analysis, maximal standardised uptake value (SUVmax) and functional volume were a predictive factor for survival. Conversely, in bivariate analysis, only functional volume was a prognostic factor. Early evaluation of treatment has been done in 4 out of 11 patients in whom PET and CT were both registered. After treatment with combined radiotherapy and chemotherapy, a negative FDG-PET/CT scan confirmed a complete long-term remission. Finally, authors suggested the use of FDG-PET/CT in ATC during initial staging. Among other imaging modalities, only preoperative CT should be requested. FDG-PET/CT could be also recommended in both early and long-term followup and in the assessment of treatment response [19].

Bogsrud et al. [20] investigated the role of FDG-PET in the management of patients with ATC. PET data were compared with other diagnostic tools (CT, ultrasound, magnetic resonance imaging, bone scan, and histology) and with clinical follow-up. In all 16 patients included, PET records resulted true positive for primary tumours. In 50% of patients, PET data influenced the clinical management. These authors concluded that FDG-PET could improve disease staging changing the clinical management of patients with ATC [20].

**Figure 2:**
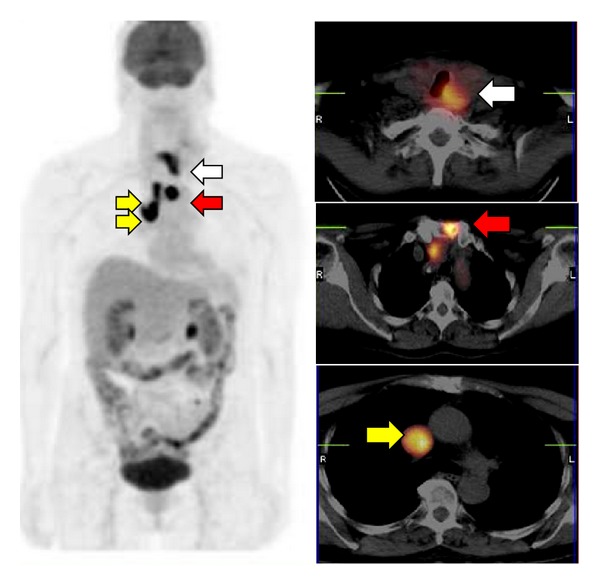
FDG-PET/CT in a 58-year-old female with ATC showing the presence of increased uptake in the thyroid tumour (white arrow) and sternal (red arrows) and mediastinal lymph nodal metastases (yellow arrows).

Overall, FDG-PET or PET/CT should be used in patients with ATC in initial staging and in the followup after surgery to evaluate metastatic disease (Figure 2). In selected cases, these functional imaging methods may be helpful in directing treatment and in evaluating the efficacy of therapy. New studies are needed to investigate the role of FDG-PET or PET/CT in detecting the proportion of DTC, ATC, and PDTC in the same tumour.

## 4. FDG-PET in Poorly Differentiated Thyroid Carcinoma (PDTC)

PDTC is an intermediate histological subtype between DTC and ATC and may be a transition form. Conversely to ATC, PDTC preserves some markers of differentiation, such as Tg and thyroid transcription factor 1 (TTF1), and does not represent a de novo tumour [26]. In dedifferentiation process, PDTC and ATC present a growing number of chromosomal alteration, such as RAS, BRAF, TP53, and b-catenin mutations [3, 11]. Activation of the PTEN/PI3 kinase/Akt/mammalian target of rapamycin pattern and mutation of the AKT or PIK3CA genes are more common in PDTC and ATC than DTC [26–29]. These metabolic pathways could be related to the different FDG-PET pattern in different subtypes of thyroid cancer.

PDTC has an intermediate GLUT1 expression and FDG uptake between ATC and DTC, because of “flip-flop” phenomenon [9, 30]. More often PDTC is an FDG-PET positive tumour [5, 9]. An in vitro study suggested that thyrotropin (TSH) increases FDG uptake in PDTC cells; so, FDG-PET scans under TSH stimulation may be more efficient [31]. Surgery and radiotherapy could be indicated in treatment of PDTC but not radioiodine treatment, because of poor radioiodine uptake.

**Figure 3:**
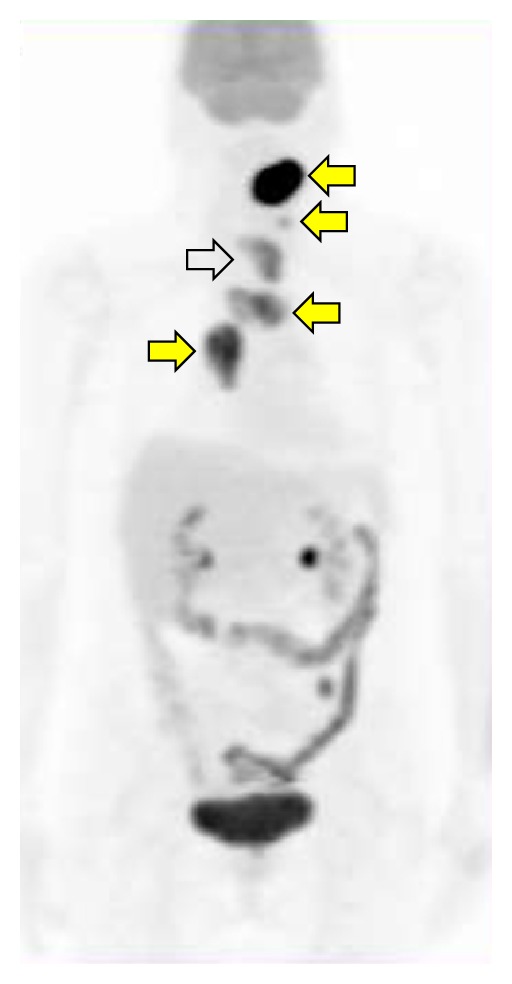
FDG-PET in a 48-year-old female with PDTC showing the presence of increased uptake in the thyroid tumour (white arrow) and multiple cervical and mediastinal lymph nodal metastases (yellow arrows).

No studies analysed the role of FDG-PET in PDCT only. Some authors suggested the use of FDG-PET or PET/CT in staging patients with PDTC (Figure 3), especially in postthyroidectomy staging of high-risk patients [11]. Some authors studied the role of FDG-PET in all thyroid cancer subtypes but included only few cases of PDTC in their analysis, not sufficient to conclude that FDG-PET is efficient for this histological tumour type.

More preclinical and clinical studies are needed about FDG-PET or PET/CT in PDTC to demonstrate the clinical usefulness of FDG-PET in PDTC.

## 5. FDG-PET in More Aggressive Histological Subtypes of DTC

Limited experience exists about the role of FDG-PET or PET/CT in patients with more aggressive histological subtypes of DTC, including case reports or small case series in patients with tall cell [32], diffuse sclerosing [33–35], solid/trabecular [36] and insular variant [37] of DTC. These articles underlines that FDG-PET or PET/CT seem to be very useful tools for the staging and restaging of such tumours.

## 6. Noniodine Concentrating Metastases of DTC

Radioactive iodine-refractory (RAIR) FDG-PET positive thyroid carcinomas represent the major cause of deaths from thyroid carcinomas and are therefore the main focus of novel target therapies. Although the majority of primary thyroid carcinomas leading to RAIR FDG-PET positive metastatic disease are PDTC, DTC can also be responsible for RAIR disease. Histologic characterization of metastases/recurrence in 70 RAIR FDG-PET positive thyroid carcinoma patients revealed that 47.1% had PDTC, 20% had tall-cell variant of papillary thyroid carcinoma, 22.9% had well-differentiated papillary thyroid carcinoma (including classic and follicular variants), 8.6% had HCTC, and 1.4% had ATC [30].

DTC presenting FDG uptake on PET scan and histological features such as necrosis should be considered aggressive differentiated cancers and FDG uptake in these tumours is highly prognostic for survival [38].

## 7. Conclusions

From this overview of the literature about the usefulness of FDG-PET or PET/CT in aggressive subtypes of thyroid tumours, we conclude the following:

- the role of FDG-PET or PET/CT in patients with HCTC is clear in initial staging or followup of invasive and metastatic tumours;
- FDG-PET or PET/CT is recommended in staging, followup, and posttreatment restaging of ATC, especially in metastatic disease, as published in ATA guidelines;
- further evaluations are needed to investigate the role of FDG-PET or PET/CT in PDTC, because of the difficulties connected to define the biological behaviour of this aggressive subtype of thyroid cancer;
- limited experience suggests the usefulness of FDG-PET or PET/CT in patients with more aggressive histological subtypes of DTC;
- DTC presenting as RAIR and FDG-PET positive should be considered aggressive tumours with poor prognosis.

## Figures and Tables

**Table 1:** Main findings about FDG-PET or PET/CT in Hürthle cell thyroid carcinoma, anaplastic thyroid carcinoma, and poorly differentiated thyroid carcinoma.

Type	Authors	Year	Device	Patients	Sensitivity	Specificity	Comments
HCTC	Pryma et al. [16]	2006	PET or PET/CT	44	95.8%	95%	FDG-PET has excellent diagnostic accuracy in HCTC, improving on CT and radioiodine scintigraphy. Intense FDG uptake is indicator of a poor prognosis. Patients with HCTC should undergo FDG-PET as part of their initial postoperative staging and periodically to screen for occult recurrence, particularly in patients with elevated serum thyroglobulin
	Lowe et al. [17]	2003	PET	12	91.6%	N.A.	HCTC demonstrates intense FDG uptake. PET improves disease detection and disease management in HCTC relative to anatomic or radioiodine imaging. FDG-PET should be recommended for the evaluation and clinical management of HCTC
	Plotkin et al. [18]	2002	PET	17	100%	60%	This study supports the efficiency of FDG-PET in the followup of HCTC

ATC	Grabellus et al. [9]	2012	PET/CT	4	100%	N.A.	ATC shows intense FDG uptake. FDG-PET/CT is an important imaging modality for ATC
	Poisson et al. [19]	2010	PET/CT	20	100%	N.A.	FDG-PET/CT appears to be the reference imaging modality for ATC at initial staging and seems promising in the early evaluation of treatment response and followup
	Bogsrud et al. [20]	2008	PET	16	100%	N.A.	FDG-PET may improve disease detection and have an impact on the management of patients with ATC relative to other imaging modalities

PDTC	Grabellus et al. [9]	2012	PET/CT	22	86.3%	N.A.	PDTC shows intermediate FDG uptake between DTC and ATC. FDG-PET/CT is an important imaging modality for PDTC

Legend: N.A.: not available; DTC: differentiated thyroid carcinoma; PDTC: poorly differentiated thyroid carcinoma; ATC: anaplastic thyroid carcinoma; HCTC: Hürthle cell thyroid carcinoma.
